# CLE Peptide Signaling and Crosstalk with Phytohormones and Environmental Stimuli

**DOI:** 10.3389/fpls.2015.01211

**Published:** 2016-01-07

**Authors:** Guodong Wang, Guohua Zhang, Mengyao Wu

**Affiliations:** Key Laboratory of Ministry of Education for Medicinal Plant Resource and Natural Pharmaceutical Chemistry, National Engineering Laboratory for Resource Developing of Endangered Chinese Crude Drugs in Northwest of China, College of Life Sciences, Shaanxi Normal UniversityXi’an, China

**Keywords:** CLE peptide, phytohormone, crosstalk, *Arabidopsis*, receptor

## Abstract

The CLE (CLAVATA3/Endosperm surrounding region-related) peptide family is one of the best-studied secreted peptide families in plants. Accumulated data have revealed that *CLE* genes play vital roles on stem cell homeostasis in different types of meristems. Additionally, *CLE* genes have been found to perform various biological roles in plant growth and development, and in response to environmental stimuli. With recent advances on our understanding of CLE peptide function, it is showing that the existence of potential crosstalks of CLE peptides with phytohormones and external stimuli. Complex interactions exist in which CLE petides coordinate with hormones to regulate plant growth and development, and in response to external stimuli. In this article, we present recent advances in cell-cell communication that is mediated by CLE peptides combining with phytohormones and external stimuli, and suggest additional *Arabidopsis CLE* genes that are likely to be controlled by hormones and environmental cues.

## Introduction

Phytohormones, as well as secreted peptides, are important in mediating intercellular communications to regulate numerous developmental and physiological activities, and respond to environmental cues (Betsuyaku et al., 2011; Murphy et al., 2012). The CLE (CLAVATA3/Endosperm surrounding region-related) peptide family is one of the best-studied secreted peptide families in plants. Over recent years, it is suggested that CLE peptide signaling is integrated with phytohormone signaling and is involved in responding to environmental cues to modulate a wide range of biological processes.

*CLE* genes are known to encode small, secreted peptides with a conserved C-terminal CLE motif (Cock and McCormick, 2001). The mature CLE peptides are cleaved from their precursor proteins after post-translational modification in the CLE motif such as hydroxylation and glycosylation (Matsubayashi, 2011). *CLE* genes have been identified in many plant species and some plant parasitic nematodes. In *Arabidopsis*, the CLE family comprised of 32 members, yet only a few *CLE* genes have been functionally characterized (Betsuyaku et al., 2011; Murphy et al., 2012). To date, CLE peptides have been implicated in the regulation of seed development, vascular formation, lateral root establishment, and the stem cell homeostasis in the shoot apical meristem (SAM), the root apical meristem (RAM) and (pro-)cambium (Czyzewicz et al., 2013; Ingram and Gutierrez-Marcos, 2015). Additionally, CLE peptides have been found to mediate responses to environmental stimuli including a notable role in sensing nitrate and controlling nodulation in legumes (Miyawaki et al., 2013). It is commonly recognized that CLE peptides are perceived by leucine-rich repeat receptor-like kinases (LRR-RLKs), forming the evolutionarily conserved CLE-RLK module to convey extracellular and intracellular signaling cascades (Betsuyaku et al., 2011; Murphy et al., 2012). Despite the large number of LRR-RLKs in plants, only a limited number of peptide-receptor pairs have been identified and assigned functionality. It is becoming increasingly apparent that CLE peptides are involved in various processes to establish, regulate and maintain plant development, and to respond to external stimuli.

In this perspective article, we focus on the characterization of CLE signaling pathways that integrating with phytohormone signaling and mediation of environmental stimuli to coordinate internal and external signals. We summarize studies that highlight the interactions of CLE peptides with hormones and external cues, and suggest additional *Arabidopsis CLE* genes that are likely to be regulated by phytohormones and/or environmental stimuli.

## Orchestration of CLE Peptide Signaling and Phytohormone Signaling

**FIGURE 1:**
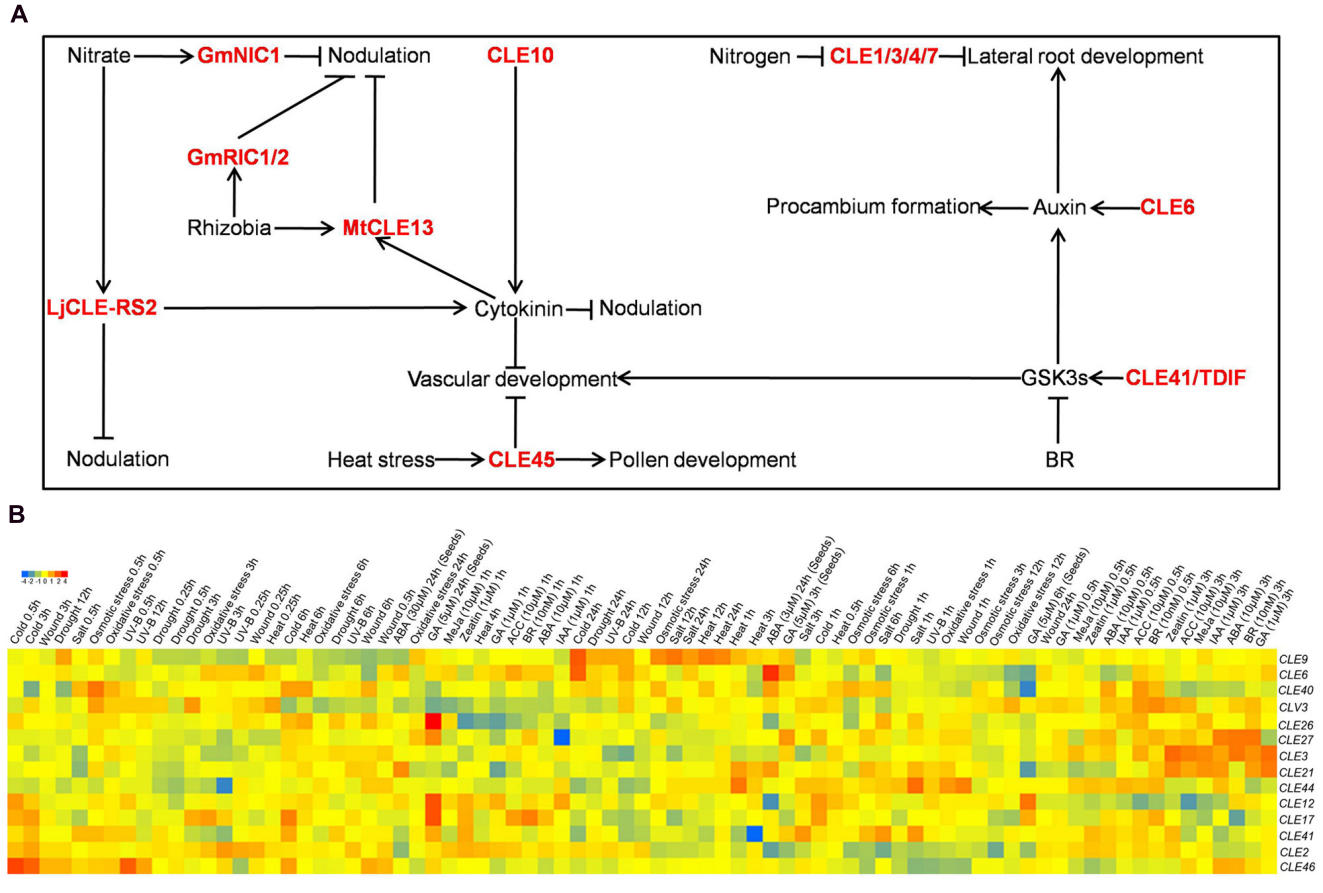
**The crosstalks of CLE peptides with diverse factors. (A)** A schematic representation of the complex interactions of CLE peptides with external and internal factors. The arrow indicate positive, while barred line indicate negative effect. CLE peptides are indicated in red. **(B)** The expression pattern of *AtCLE* upon hormones and selected stresses. The microarray data were obtained from AtGenExpress initiative. A detail description of samples and experimental design would be found at https://www.arabidopsis.org/portals/expression/microarray/ATGenExpress.jsp. Gene expression is displayed as normalized log2-transformed values, which is visualized by the color scale.

It has revealed that CLE peptide signaling integrated with phytohormone signaling to control various biological processes in plants (**Figure 1A**; **Table 1**). CLE6 and CLE41/TDIF activated auxin transcriptional reporters and transporters including DR5pro:GUS, IAA2pro:GUS, PIN1pro:GUS, and PIN3pro:GUS, suggesting induction of an immediate auxin response upon CLE peptides (Whitford et al., 2008). In addition, CLE6 potentiated an effect of CLE41/TDIF on promoting procambium proliferation. Furthermore, the effect was synergistically strengthened in the presence of a synthetic auxin NAA, and weakened in the presence of an auxin polar transport inhibitor NPA (Whitford et al., 2008). Consistently, the CLE41/TDIF peptide-induced procambium proliferation was abolished by a mutation in the *Monopteros/ARF5* gene, an auxin response factor required for mediating auxin stimuli (Whitford et al., 2008). Altogether, these results indicate that vascular patterning is regulated by different CLE peptides in conjunction with auxin signaling.

**Table 1:** A summary of crosstalks between CLE peptides with hormones and environmental stimuli.

CLE peptide	Species	Factor(s) interacted	Function(s)	Reference(s)
CLE1/3/4/7	*Arabidopsis thaliana*	Nitrogen	Inhibiting later root growth	Araya et al., 2014
CLE6	*A. thaliana*	Auxin/GA	Promoting procambium proliferation; compensation for GA deficiency	Whitford et al., 2008; Bidadi et al., 2014
CLE10	*A. thaliana*	Cytokinin	Inhibiting protoxylem vessel formation	Kondo et al., 2011
CLE14/20	*A. thaliana*	Cytokinin	Inhibiting root growth	Meng and Feldman, 2010
CLE26	*A. thaliana*	Auxin	Regulating root architecture and protophloem formation	Czyzewicz et al., 2015
CLE40	*A. thaliana*	ABA/auxin/cytokinin	Promoting ABA biosynthesis and signaling, repressing cytokinin signaling and differentially regulating auxin signaling	Pallakies and Simon, 2014
CLE41/TDIF	*A. thaliana*	Auxin/BR	Later root development; xylem vessel formation	Cho et al., 2014; Kondo et al., 2014
CLE45	*A. thaliana*	Heat stress	Pollen growth and seed production	Endo et al., 2013
OsCLE48	*Oryza sativa*	Auxin	N/D	Guo et al., 2015
LjCLE-RS1/2	*Lotus japonicus*	Rhizobia/cytokinin/nitrate	Inhibiting nodule development	Okamoto et al., 2009; Okamoto et al., 2013; Sasaki et al., 2014
LjCLE19/20	*L. japonicus*	Phosphate	N/D	Funayama-Noguchi et al., 2011
MtCLE12/13	*Medicago truncatula*	Rhizobia	Inhibiting nodule development	Mortier et al., 2010; Mortier et al., 2012
GmRIC1/2	*Glycine max*	Rhizobia	Inhibiting nodule development	Reid et al., 2011
GmNIC1	*G. max*	Nitrate	Partially inhibiting nodulation	Reid et al., 2011

*N/D, not determined*.

In addition to the interaction between CLE41/TDIF and auxin, CLE41/TDIF, along with brassinosteroids (BR), was found to determine the xylem vessel formation by regulating the GSK3s activity (Kondo et al., 2014). CLE41/TDIF prevents the differentiation of procambial cells into xylem cells, whereas BR positively regulates xylem differentiation (Hirakawa et al., 2008; Clouse, 2011). It has been shown that TDR/PXY, the CLE41/TDIF receptor, interacts with GSK3s, and activates GSK3s in a CLE41/TDIF-dependent manner. BES1, as a downstream target of GSK3s, mediates CLE41/TDIF-TDR-GSK3s signaling to suppress xylem differentiation (Kondo et al., 2014). Additionally, CLE41/TDIF-initiated TDR signaling activates BIN2, which further interacts with auxin signaling by phosphorylating ARF7 and ARF19 to inhibit their interactions with AUX/IAAs and positively regulates their target genes *LBD16* and *LBD29* to modulate the lateral root development (Cho et al., 2014). However, as BR exerts no effect on regulation of BIN2 activity, it suggests an immediate CLE41/TDIF-induced regulation of BIN2 in lateral root formation (Cho et al., 2014). This provides an example of complex interaction among CLE41/TDIF, BR and auxin signaling to regulate root development.

The CLE10 peptide, similar to cytokinin, suppressed protoxylem formation in *Arabidopsis* roots, implying a possible crosstalk between CLE peptide and cytokinin. Further investigation revealed the expression of *ARR5* and *ARR6*, two negative regulators of cytokinin signaling, was repressed by CLE10 (Kondo et al., 2011). Consistently, protoxylem formation of the lateral root, but not the primary root, was inhibited in *arr5 arr6* double mutants despite no alteration is observed in either single mutant. Intriguingly, the *clv2* mutant exhibited insensitivity to CLE10 peptides in the suppression of protoxylem formation, suggesting that CLE10 acts through CLV2 to regulate the protoxylem formation (Kondo et al., 2011). Additionally, ARR10 and ARR12, two positive regulators of cytokinin signaling, were shown to be necessary for CLE10 induced protoxylem inhibition as the *arr10 arr12* double mutant was unresponsive to the CLE10 peptide (Kondo et al., 2011). It is therefore suggested that CLE10 suppresses the expression of *ARR5* and *ARR6*, by which results in promoting cytokinin signaling to inhibit protoxylem formation.

*In vitro* application of either CLE14 or CLE20 peptides, or overexpression of *CLE14* and *CLE20* resulted in short-root phenotype by reducing cell division rates in the RAM (Meng and Feldman, 2010). The short-root phenotype caused by the exogenous application of the CLE14 or CLE20 peptide cannot be overcome by auxin or cytokinin treatment, while cytokinin partially rescued the short-root phenotype induced by overexpression of *CLE14* or *CLE20 in planta* (Meng and Feldman, 2010). This result implies that cytokinin, but not auxin, may influence CLE14/CLE20 functions by affecting the post-translational regulation of CLE peptides *in vivo*, probably resulting in an alteration in the availabilities and/or abundances of CLE14/CLE20 peptides. Alternatively, overexpression of *CLE14* or *CLE20* may down-regulate cytokinin biosynthesis gene(s) or promote cytokinin metabolic gene(s) which can be compensated by exogenous cytokinin. However, the molecular mode of action of crosstalk between CLE and cytokinin in this process awaits elucidation.

As reported recently, in additional to CLE45, CLE26 affected primary root protophloem (Czyzewicz et al., 2015). *CLE26* is expressed in the stele at the phloem pole. Expression of *CLE26* is increased significantly upon auxin treatment, indicating a possible interaction between CLE26 and auxin (Czyzewicz et al., 2015). Further studies revealed that exogenously applied CLE26 peptide resulted in altered auxin responses as evidenced by reduced auxin response marker DR5pro:GUS and elevated auxin sensor DII:VENUS. In addition, pPIN1::PIN1:GFP is reduced in the presence of CLE26 peptide, although no effect on the *PIN1* gene expression. This indicates that CLE26 influences auxin signaling through modulating the activity of the polar auxin transporter (Czyzewicz et al., 2015). Collectively, the CLE26 peptide represses the distribution and/or abundance of auxin in the RAM, possibly by decreasing the abundance of PIN1 through post-translational regulation. However, the biological significance of auxin alteration induced by CLE26 is unclear. Nevertheless, it is also intriguingly to unravel the mechanism by which auxin transcriptionally regulates the *CLE26* and the biological consequences by elevating its transcripts.

It has been shown that CLE40 integrated with phytohormone pathways by regulating hormone synthesis, signaling, and their target genes (Pallakies and Simon, 2014). Genes involving in abscisic acid biosynthesis and signaling were down-regulated in *cle40* mutants, whereas auxin-related genes were differentially expressed. CLE40 represses cytokinin signaling by supressing the expression of key genes in cytokinin signaling and biosynthesis (Pallakies and Simon, 2014). Altogether, it is suggested that CLE40 modulates phytohormone signaling in distinct modes through multi-pronged targets that comprise phytohormone synthesis, signaling, and downstream genes.

The application of gibberellin (GA) promoted *CLE6* expression, suggesting a direct and long-distant regulation of *CLE6* by GA hormone. Conversely, application of CLE6 peptide exerted no effect on the growth and development of GA-deficient mutant plants (Bidadi et al., 2014). However, over-expression of *CLE6* in a GA-deficient mutant partially rescued the mutant phenotype, suggesting that CLE6 could compensate for the GA deficiency. Grafting of GA-deficient mutant plants to 35S::CLE6 transgenic plants complemented the shoot phenotype associated with GA deficiency, suggesting CLE6 can exert its action over a long distance (Bidadi et al., 2014). However, whether the CLE6 peptide itself moves through the vascular system is yet to be demonstrated.

Forty-seven *CLE* genes have been identified from rice (Kinoshita et al., 2007). OsCLE48, a rice CLE peptide, is significantly induced by auxin (Guo et al., 2015). *OsCLE48* rescued the *clv3-2* mutant phenotype when driven by the native *CLV3* promoter. However, overexpression of *OsCLE48* in rice failed to alter the shoot development (Guo et al., 2015), implying the functional divergence of *CLE* genes in *Arabidopsis* and rice. It is still unclear that function of OsCLE48 and the biological consequence of transcriptionally regulating of *OsCLE48* by auxin.

## CLE Peptide Signaling Upon Environmental Stimuli

Plants are continuously exposed to a wide range of environmental stimuli. Considerable advances have been made in our understanding of interactions between CLE peptides and environmental stimuli (**Figure 1A**; **Table 1**). A study on CLE45 peptide provided an example of a CLE peptide mediating the signal of an environmental cue. In addition to its role on inhibiting protophloem differentiation via BAM3 in roots, CLE45 is implicated in pollen-pistil interaction upon heat stress (Depuydt et al., 2013; Endo et al., 2013). CLE45 is preferentially expressed in the stigma at normal temperature, whereas its expression domain expand into the transmitting tract at elevated temperature, suggesting a temperature-dependent function of CLE45 (Endo et al., 2013). Two RLKs, SKM1 and SKM2, are expressed preferentially in pollen and pollen tubes. Additionally, pollen tube growth of the *skm1 skm2* double mutant displayed complete insensitivity to CLE45 peptide, suggesting that SKM1 and SKM2 may function as receptors of CLE45 in this process (Endo et al., 2013). This was confirmed by a genetic study showing that *skm1 skm2* double mutants phenocopied the CLE45-RNAi plants. Furthermore, a direct and specific binding of CLE45 peptide with SKM1 protein was demonstrated (Endo et al., 2013). In conclusion, CLE45 mitigates heat stress by binding with SKM1/SKM2 to sustain pollen growth under higher temperatures and maintain successful seed production.

It is known that lateral roots stop growing under severe deficiency of nitrogen (N), while the expression of *CLE1*/*3/4/7* were induced under N-deficient conditions (Araya et al., 2014). The *clv1* mutant exhibited progressive growth of lateral roots under N-deficient conditions. Conversely, overexpression of *CLE1*/*3/4/7* repressed the emergence and growth of lateral roots. However, this inhibitory action of *CLE3* was abolished in the *clv1* mutant (Araya et al., 2014). *CLE1*/*3/4/7* are predominantly expressed in the root pericycle, while the location of CLV1 is restricted in phloem companion cells (Araya et al., 2014). Altogether, these results indicate that CLV1 mediates a N-responsive CLE peptide signaling pathway that negatively regulates lateral root development under N-deficient conditions.

Funayama-Noguchi et al. (2011) identified two *LjCLE* genes, *LjCLE19* and *LjCLE20*, which respond specifically in the presence of phosphate (Pi). *LjCLE19* and *LjCLE20* were up-regulated specifically and significantly upon excess Pi. Along with the increase in Pi level, expressions of *LjCLE19* and *LjCLE20* increased prior to the increment of Pi content in plants. However, the Pi content in plants decreased prior to the reduction of *LjCLE19* and *LjCLE20* expression with external Pi decreased (Funayama-Noguchi et al., 2011). Nevertheless, it remains largely unknown how exactly LjCLE19 and LjCLE20 mediated the phosphate signaling to modulate plant growth and development.

## CLE Peptide Signaling in Nodulation

It is well known that many CLE peptides play vital roles in legume-rhizobium symbioses (Miyawaki et al., 2013). A search of the *Lotus japonicus* genome database has identified 39 *LjCLE* genes, among which *LjCLE-RS1* and *LjCLE-RS2* are significantly up-regulated in rhizobial inoculated roots (Okamoto et al., 2009). HAR1 encodes a LRR-RLK which is highly similar to CLV1. The *har1* mutant exhibits a hypernodulation phenotype (Krusell et al., 2002; Nishimura et al., 2002). Overexpression of *LjCLE-RS1* and *LjCLE-RS2* suppress nodule development. However, this inhibitory effect is abolished in the *har1* mutant, implying that *LjCLE-RS1* and *LjCLE-RS2* inhibit nodulation in HAR1-dependent manner (Okamoto et al., 2009). Further studies revealed that *LjCLE-RS1/2*, as long-distance signals, were root-derived signals that were recognized by HAR1 in the shoot. Once perceived, the CLE-RS1/2-HAR1 signaling activated the shoot-derived cytokinins which systemically inhibited nodulation (Okamoto et al., 2013; Sasaki et al., 2014). Constitutive activation of *LjCLE* genes incurred disappearance of auxin responses (Suzaki et al., 2012, 2013). In addition to HAR1, KLV, and LjCLV2 encode a RPK2-like RLK and a CLV2-like protein, respectively (Miyazawa et al., 2010; Krusell et al., 2011). Genetic and biochemical studies revealed that HAR1 and KLV function in the same pathway via forming a receptor complex by which *LjCLE-RS1* and *LjCLE-RS2* signaling are transmitted (Krusell et al., 2011). Similarly, mutations in *LjCLV2* result in increased nodulation (Miyazawa et al., 2010). However, it is not known by what mechanism LjCLV2 controls the nodulation. Furthermore, *LjCLE-RS2* is strongly up-regulated upon excess nitrate application in roots, suggesting that LjCLE-RS2 plays key roles in nitrate sensing (Okamoto et al., 2009). It is known that accumulation of nitrate suppresses nodulation. Thus, *LjCLE-RS2* may sense the nitrate to negatively regulate nodulation.

MtCLE12 and MtCLE13, two MtCLEs identified from *Medicago truncatula*, were also implicated during nodulation (Mortier et al., 2010). *MtCLE12* and *MtCLE13* were up-regulated in nodulated roots, whereas the expression of *MtCLE13* increased much earlier than that of *MtCLE12*. Moreover, *MtCLE13* expression was induced by cytokinin, while that of MtCLE12 was unaffected (Mortier et al., 2010, 2012). Nevertheless, the CLE domain sequences of MtCLE12 and MtCLE13 are highly similar to the CLE domains of LjCLE-RS1 and LjCLE-RS2, suggesting that MtCLE12/MtCLE13 and LjCLE-RS1/LjCLE-RS2 may exert a comparable function during nodulation. Indeed, the ectopic expression of *MtCLE12* and *MtCLE13* inhibited nodulation, which was mediated by a LRR-RLK, SUNN (Schnabel et al., 2005; Mortier et al., 2010).

Reid et al. (2011) identified three CLE peptides, namely GmRIC1, GmRIC2, and GmNIC1, in soybean. The expression of *GmRIC1* and *GmRIC2* were induced by rhizobial inoculation, while the expression of *GmNIC1* was up-regulated by nitrate (Reid et al., 2011). Overexpression of *GmRIC1* and *GmRIC2* inhibited soybean nodulation systemically and required the presence of GmNARK which encodes a LRR-RLK (Searle et al., 2003; Reid et al., 2011; Lim et al., 2011). In contrast, overexpression of *GmNIC1* partially reduced nodulation locally, which also required GmNARK (Reid et al., 2011). These results suggest the requirement of GmNARK, possibly as the receptor for GmRIC1/2 and GmNIC1, in both inoculation- and nitrate-induced regulation of nodulation in soybean.

In summary, CLE peptides play critical roles in nodulation formation in legume. A recurrent theme in CLE-mediated nodulation formation is the requirement of a LRR-RLK to perceive the CLE peptide signaling, suggesting evolutionary conservation and commonality in the regulation of nodulation. In addition, *LjCLE-RS2* and *GmNIC1* are nitrate-responsive, while *MtCLE13* expression is induced by cytokinin, implying a greater level of complexity in the interactions between CLE peptides, hormones and external stimuli on controlling nodulation.

## The Expression of Many *Arabidopsis CLE* Genes is Perturbed by Phytohormones and Environmental Stimuli

Microarray datasets allowed us to identify *Arabidopsis CLE* genes that are regulated by phytohormones and environmental stimuli. *In silico* expression data for 14 out of 32 *Arabidopsis CLE* genes are available for further analysis (Kilian et al., 2007; Goda et al., 2008). Notably, *CLE3* is dramatically up-regulated by all tested hormones correlating with the time course, while its expression was repressed by cold, salt and UV-B (**Figure 1B**). *CLE2* is generally inhibited by hormones and external stresses under sustained treatment, and induced under short-term treatment. The expression of *CLE9* is not significantly induced by hormones, but was dramatically induced by almost all selected stresses under sustained treatment (**Figure 1B**). *CLV3*, *CLE2*, *CLE3*, *CLE21*, and *CLE46* exhibit diverse responses upon hormones and abiotic stresses. Much to our surprise, *CLV3*, known as a key player in regulation of stem cell homoeostasis, is shown not only respond to hormones, but also display variable responses to selected stresses (**Figure 1B**). In contrast, *CLE12*, *CLE17*, *CLE26*, *CLE27*, *CLE40*, *CLE41*, and *CLE44* remain unchanged, with the exception of *CLE27* which is prominently repressed by auxin (**Figure 1B**). Collectively, these data suggest that *Arabidopsis CLE* genes are distinctly perturbed by phytohormones and environmental stimuli depending on time of treatment and concentration. The challenge will be to develop ways to attribute specific functions to these *CLE* genes that are transcriptionally regulated by hormones and stresses, and to understand how exactly they are controlled and their roles in adaptive plant growth.

## Conclusion and Perspectives

Over recent years, crosstalks between CLE peptides with phytohormones and environmental cues have been shown (**Figure 1A**; **Table 1**). Clearly, concluding from the examples presented, one could speculate that there are complicated and diverse regulatory networks involved in these crosstalks, but most await further elucidation. Regarding the functional dissection of *CLE* genes, antagonistic peptide technology and CRISPR gene editing technology may be helpful to overcome functional redundancies and difficulties in obtaining loss-of-function mutants (Song et al., 2013; Ma et al., 2015). CLE peptides are perceived by the extracellular domains of RLKs, but information on peptide–receptor pairs with assigned functions is scarce. The identification and characterization of peptide-receptor pairs will continue to yield insights into how they are integrated with diverse factors. To this end, it is essential to understand the spatial and temporal control of *CLE* gene expression in various plant species, as has been shown for many *CLE* genes in *Arabidopsis* (Jun et al., 2010). It is also important to identify internal and external factors that regulate the expression of *CLE* genes. As a first step, it is shown that many *Arabidopsis CLE* genes are perturbed by phytohormones and environmental stimuli (**Figure 1B**), which open avenues to gain insights into the crosstalk of *Arabidopsis* CLE peptides with diverse factors. The future challenge will be to develop ways to attribute specific functions to these responsive *CLE* genes, and ultimately match their functions to the supposed complex regulatory networks that integrating with phytohormones and/or environmental stimuli.

## Author Contributions

GW conceived and wrote the manuscript; GZ and MW critically reviewed the manuscript.

## Conflict of Interest Statement

The authors declare that the research was conducted in the absence of any commercial or financial relationships that could be construed as a potential conflict of interest.
